# Acute Portal Vein Thrombosis as an Initial Presentation of Protein C Deficiency: A Case Report

**DOI:** 10.7759/cureus.40407

**Published:** 2023-06-14

**Authors:** Ekaterina Proskuriakova, Ranjit B Jasaraj, Dhan B Shrestha, Vijay Ketan Reddy, Pam Khosla

**Affiliations:** 1 Internal Medicine, Mount Sinai Hospital, Chicago, USA; 2 Hematology and Oncology, Mount Sinai Hospital, Chicago, USA

**Keywords:** pulmonary emboli, superior mesenteric vein thrombosis, protein c and protein s deficiencies, portal vein tumor thrombosis, protein c

## Abstract

Protein C (PC) is an essential vitamin K-dependent protein that regulates thrombosis and hemostasis in the body. A mutation in the PROC gene on chromosome 2q14.3 results in PC deficiency. The clinical presentation of PC deficiency can vary, ranging from a single vein thrombosis to disseminated intravascular coagulation, purpura fulminans, or even life-threatening complications such as sepsis. Here, we present a case of a 37-year-old female who was found to have acute portal vein thrombosis as an initial presentation of PC deficiency. She presented to the hospital with acute onset of abdominal pain associated with nausea, blood-streaked emesis, and bloody bowel movement.

## Introduction

Protein C (PC) is an essential vitamin K-dependent protein that regulates thrombosis and hemostasis in the body. It was first found and described in 1976 by Stenflo [1]. The thrombin activates PC and becomes activated PC (APC), inhibiting activation of the membrane-bound activated factors V and VIII by degrading specific arginine residues [2]. The incidence of severe forms (homozygous or compound heterozygous) of PC deficiency is approximately one in 5,00,000 people, while the incidence of heterozygous PC is not that rare, with one in 500 births [3].

A mutation in the PROC gene on chromosome 2q14.3 results in PC deficiency. The risk of developing thrombosis among individuals will vary significantly depending on genetic and environmental factors [4]. PC deficiency can present as thrombosis, purpura fulminans, or even life-threatening complications such as sepsis [5]. PC deficiency can be congenital (heterozygous or homozygous) and acquired. Heterozygous patients who develop venous thromboembolism (VTE) usually have some other risk factors like the use of oral contraceptives, pregnancy, recent surgery, or immobilization that can lead to VTE [6]. 

Portal vein thrombosis (PVT), an acute or chronic portal vein blood flow obstruction, can be a rare presentation of PC deficiency. Very few studies describe PVT as a complication of PC deficiency due to a low incidence of less than 0.004%. [7]. Patients with PVT can present with different clinical features, such as intestinal congestion, ischemia, abdominal pain, rectal bleeding, or vomiting [8].

Here, we present a case of a 37-year-old female who was found to have acute PVT as an initial presentation of PC deficiency. She presented to the hospital with acute onset of abdominal pain associated with nausea, blood-streaked emesis, and bloody bowel movement.

## Case presentation

A 37-year-old female with a past medical history of migraines presented with severe epigastric pain for one week that had progressively worsened. Her family history was significant for recurrent deep vein thrombosis (DVTs) in her mother. Her mother has never had any thrombophilia workup done. A week prior, she had multiple emergency room visits for abdominal pain; the preliminary labs and abdominal imaging, such as abdominal X-ray and ultrasound, were unrevealing; and the symptoms improved after conservative management with antiemetics and antacids. 

The patient is a nonsmoker, drinks alcohol occasionally, and has never used drugs. She lives with her husband and daughter. She is a housewife. 

Her symptoms returned a day later, and she presented to the emergency room with severe 10/10 diffuse abdominal pain associated with nausea, mild hematemesis, and hematochezia. On arrival at the emergency department, she was hemodynamically stable. Labs were significant for leukocytosis with a white blood cell count (WBC) of 27 × 109/L and anemia with hemoglobin (Hgb) of 11.1 gm/dL with a mean corpuscular volume of 86 fL. Her comprehensive metabolic panel (CMP) was unremarkable (Table 1).

**Table 1:** Comprehensive metabolic panel on admission mEq/L, milliequivalents per liter; mmol/L, millimoles per liter; mg/dL, milligrams per deciliter; U/L, units per liter

	On admission	Range
Sodium	136 mEq/L	133-134 mEq/L
Potassium	4.3 mEq/L	3.5-5.2 mEq/L
Chloride	106 mEq/L	98-107 mEq/L
Anion gap	10 mmol/L	4-11 mmol/L
Calcium	9.0 mg/dL	8.6-10.3 mg/dL
Glucose	89 mg/dL	70-99 mg/dL
Creatinine	0.4 mg/dL	0.6-1.2 mg/dL
BUN	14 mg/dL	7-25 mg/dL
BUN/creatinine ratio	36	5-19
Total protein	6.9 mg/dL	6.4-8.9 mg/dL
AST	44 U/L	13-39 U/L
ALT	60 U/L	7-52 U/L
Lactic acid	0.8 mmol/L	0.5-2.0 mmol/L

**Figure 1:**
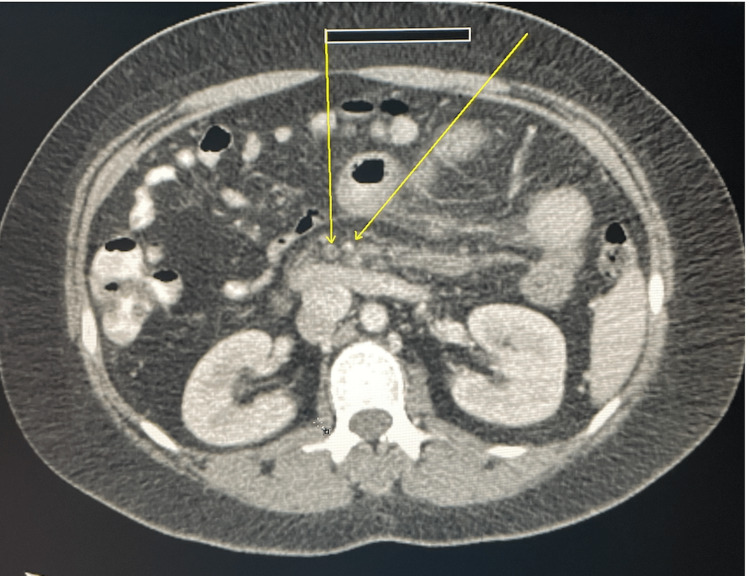
Thrombosis of the portal vein and superior mesenteric vein

**Figure 2:**
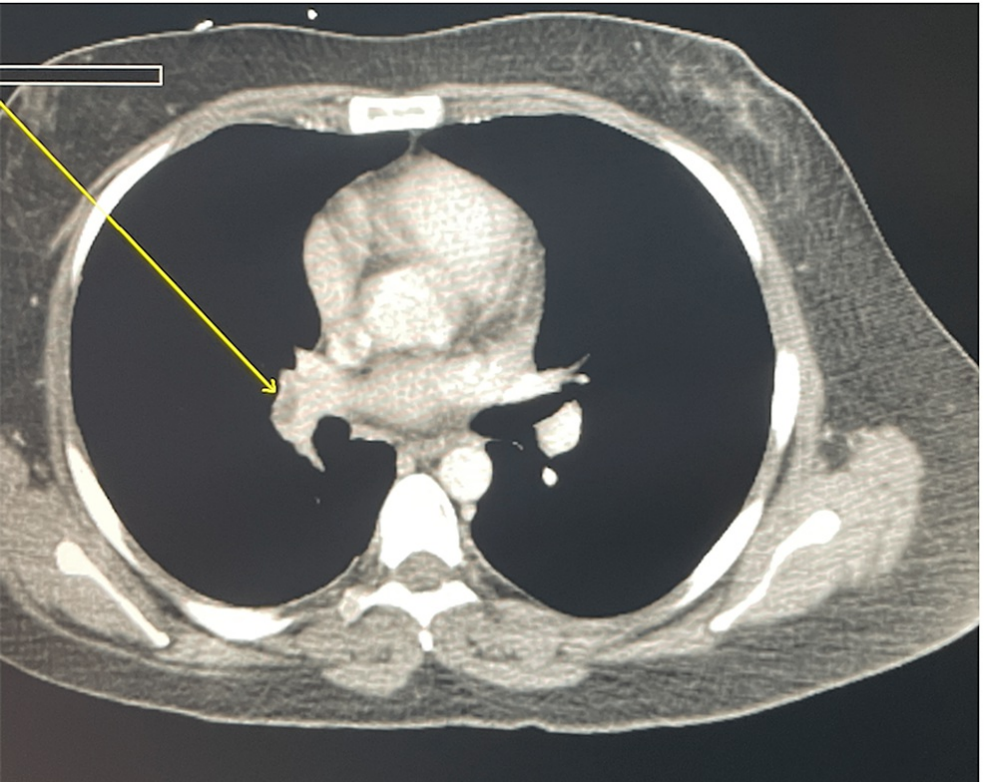
CT pulmonary angiogram: pulmonary emboli

Computer tomography (CT) of the abdomen and pelvis revealed acute thrombosis of the portal vein and SMV, ischemic enteritis associated with moderate regional mesenteric vascular engorgement and mesenteric edema, and pulmonary emboli seen along the imaged lower lung zones (Figures 1, 2). Lactic acid was normal all her hospital stay (Table 2). Ultrasound showed normal echogenicity and echotexture of the liver, and no masses were detected.

**Table 2:** Lactic acid laboratory results during the first five days mmol/L, millimoles per liter

Hospital day	Lactic acid
Day 1	1.1 mmol/L
Day 2	1.2 mmol/L
Day 3	0.9 mmol/L
Day 4	0.7 mmol/L
Day 5	0.6 mmol/L

Venous duplex showed no evidence of acute lower extremity deep venous thrombosis. The patient was initiated on a heparin drip, and vascular surgery and hematology were consulted.

There was a suspicion of Mallory-Weiss tear in the setting of retching, vomiting, and a high possibility of bleeding from esophageal and gastric varices secondary to PVT and mesenteric vein thrombosis. As there was no evidence of esophageal or gastric varices on the CT scan, esophagogastroduodenoscopy (EGD) was not done during this hospital stay. The patient was kept nothing per os, nasogastric tube suctioning, and started on proton pump inhibitors IV twice daily, hydromorphone for pain, ceftriaxone, and metronidazole. CT pulmonary angiography has demonstrated embolism at the segmental pulmonary artery level, no features of right heart strain, and moderate volume right pleural effusion with atelectasis at the right lower lobe. Echo demonstrated a normal ejection fraction of 60-65% with no right heart strain. She was monitored closely for signs of bleeding; surgery was deferred, given the extensiveness of the surgical intervention and the possibility of affecting her quality of life. A complete thrombophilia profile, including factor V Leiden, prothrombin gene mutation, antiphospholipid antibody, and PC activity functions, was performed, which revealed a PC antigen level of 54% (with the normal level being 70-150%) and activity of 37 IU dL^−1^ (with normal range of 65­-135 IU dL^−1^), which is considered to be low. The repeat PC antigen and PC gene test were planned for later on an outpatient visit, approximately three months after starting the treatment, but the patient did not come to the clinic. 

Her symptoms improved over a week. Over that time, she has completed a course of antibiotics of ceftriaxone and metronidazole for ischemic colitis. She remained on a heparin drip for 10 days while in the hospital due to the opportunity to reverse anticoagulation if the patient developed hematemesis again. She subsequently was switched to apixaban 10 mg twice daily for seven days, followed by 5 mg twice daily upon discharge indefinitely.

## Discussion

PVT is a condition associated with the occlusion of the portal vein or its tributaries, including the cystic and left and right gastric, paraumbilical, and prepyloric veins [9]. This condition could lead to three main complications, such as small bowel ischemia, ischemic hepatitis, and gastrointestinal bleeding [9]. PVT could be categorized into four groups: thrombosis confined to the portal vein beyond the confluence of the splenic and SMV, extension of the thrombus into the SMV but with patent mesenteric vessels, diffuse thrombosis of the splanchnic venous system but with large collaterals, and extensive splanchnic venous thrombosis but with fine collaterals [10]. Our patient developed acute thrombosis of the portal vein and SMV with signs of ischemic colitis. 

The risk factors for developing PVT include cirrhosis, malignancy, mutation of the Janus kinase 2 (JAK2), and thrombophilia. The rarest thrombophilia is PC and protein S deficiency, with a prevalence of less than 1% in the general population [11]. Fisher et al. found in their research that among 29 patients with PVT, 18 of the patients, or 62% of them, had one or more than one deficiency of anticoagulant proteins, including PC. Eight of the 18 people had combined PC and protein S deficiency, nine had PC and antithrombin deficiency, and six had a combined deficit of the three proteins. Patients with PVT should always be tested for thrombophilia, including PC deficiency [12]. 

In the general population, there is a balance between procoagulant and anticoagulant factors such as factors V and VIII and protein S and PC, respectively. PC is formed in the hepatocytes and circulates in the plasma as a heterodimeric complex containing a heavy and light chain. To be activated, PC requires two membrane receptors, the endothelial PC receptor and thrombomodulin, located on the surface of endothelial cells, to convert PC to APC [13]. APC inactivates procoagulant factors V and VIII via a proteolytic mechanism and promotes cytoprotective and anti-inflammatory effects [5]. 

PC deficiency can be congenital (homozygous and heterozygous) or acquired. Most patients present with heterozygous mutation in the PC gene (PROC) on chromosome 2q14.3 and present with asymptomatic clinical picture or VTE, as well as pulmonary embolism [3]. Usually, with heterozygous mutations, patients have other risk factors, such as recent surgeries or pregnancy, that can lead to VTE [6]. Our patient was innately deficient in PC and was using OCP, which could have further predisposed her to the current presentation. Patients with heterozygous mutations of the PC gene have one mutated gene and one normal gene [14]. In addition, the clinical picture varies between individuals due to the degree of deficiency of the gene defined by two laboratory tests for this condition, antigen and activity assays [15]. The test results we received from our patient showed 54% of PC antigen and 37% of PC activity, which is considered low.

Routine evaluation for hypercoagulable conditions in patients with VTE is not currently recommended. The common indications for thrombophilia testing, including PC and protein S deficiency, antithrombin, the factor V Leiden, and prothrombin gene mutations, are those patients with VTE who have first-degree family members with a history of VTE before the age of 45 years. Those individuals who do not have a family history of VTE should be tested if they developed VTE before 45 years old, if they experience recurrent thrombosis, or if those who developed VTE in unusual places such as portal, hepatic, mesenteric, or cerebral veins. [13]. 

Anticoagulant treatment is the primary therapy for acute non-cirrhotic PVT and should be started at the diagnosis [16]. Low-molecular-weight heparin (LMWH) or unfractionated heparin is preferred. In their retrospective study, Amitrano et al. revealed that in acute splanchnic vein thrombosis, anticoagulants effectively achieved recanalization in 45% of patients and secured patients from recurring thrombosis in the future if they are given all their life continuously [17]. Current guidelines recommend treating PVT for at least six months [16]. However, the underlying mechanism of thrombus production might require indefinite treatment [18]. Our patient was started on an unfractionated heparin drip, which she continued to receive for 10 days while in the hospital due to the high chance of bleeding. On discharge, she transitioned to apixaban 10 mg twice daily for seven days, followed by 5 mg twice daily. A recent study by Janczak et al. demonstrated the successful use of direct-acting oral anticoagulants (DOAC) in patients with venous thrombosis of atypical locations such as portal and mesenteric [19]. DOACs do not need continuous routine monitoring of the coagulation profile or daily subcutaneous injections of the medication, making them convenient and comfortable to use and increasing patient compliance [20]. It is recommended that the patients have regular follow-ups with the primary care provider or hematology.

## Conclusions

Protein vein thrombosis is one of the indicators for thrombophilia workup, including PC deficiency. The clinical manifestation of VTE depends on the degree of deficiency of the PC defined by antigen and activity assays. Abnormal PC results should be repeated at least three months after treatment initiation. Anticoagulant treatment is the primary therapy and should be started at the diagnosis of thrombosis. DOACs could be used in patients with acute venous thrombosis in atypical locations like PVT.
